# Current Status of Immunotherapy for Localized and Locally Advanced Renal Cell Carcinoma

**DOI:** 10.1155/2019/7309205

**Published:** 2019-04-01

**Authors:** Fady Ghali, Sunil H. Patel, Ithaar H. Derweesh

**Affiliations:** Department of Urology UC San Diego School of Medicine, La Jolla, California, USA

## Abstract

Systemic therapy strategies in the setting of localized and locally advanced renal cell carcinoma (RCC) have continued to evolve in two directions: as adjuvant therapy (to reduce risk of recurrence or progression in high risk localized groups), or as neoadjuvant therapy as a strategy to render primary renal tumors amenable to planned surgical resection in settings where radical resection or nephron-sparing surgery was not thought to be safe or feasible. In the realm of adjuvant therapy, the results of phase III randomized clinical trials have been mixed and contradictory; nonetheless based on the findings of the landmark S-TRAC study, the tyrosine kinase inhibitor Sunitinib has been approved as an adjuvant agent in the United States. In the realm of neoadjuvant therapy, presurgical tumor reduction has been demonstrated in a number of phase II studies utilizing targeted molecular agents. The advent of immunomodulation through checkpoint inhibition as first line therapy for metastatic RCC represents an exciting horizon for adjuvant and neoadjuvant strategies. This article reviews the current status and future prospects of adjuvant and neoadjuvant immunotherapy in localized and locally advanced RCC.

## 1. Introduction

Renal Cell Carcinoma (RCC) is common cancer globally, with approximately 400,000 people being diagnosed with RCC in 2018, a notable increase in incidence rates with time, and is among the top ten most common malignancies in the United States [1, 2]. Due to the widespread use of cross-sectional imaging, incidence of RCC has increased with most cases presenting as localized disease [3–5]. Despite such stage migration, the risk of recurrence remains high [6–9]. Poor prognosis of patients with recurrence and the risks associated with locally advanced resection or nephron-sparing surgery in the imperative setting for complex masses have served as an impetus to explore further approaches to improve outcomes.

The improved response rates and outcome in metastatic RCC ushered in the era of targeted therapies; both tyrosine kinase inhibitor (TKI) therapy and immune checkpoint inhibitors (ICI) have stimulated investigation into the utility of these agents as adjuvants in the setting of localized and locally advanced disease to reduce the risk of recurrence and improve survival [10–15]. Herein, we review and summarize the current status and future directions of adjuvant and neoadjuvant immunotherapeutic strategies in localized and locally advanced RCC, focusing on current literature and ongoing clinical trials in both areas.

## 2. Methodology

### 2.1. Literature Search

PubMed, MEDLINE, Cochrane Central Register of Controlled Trials, the American Society of Clinical Oncology, and ClinicalTrials.gov were searched with keywords including “neoadjuvant”, “adjuvant”, “immunotherapy”, “targeted therapy”, “immune checkpoint (anti-PD-1) inhibitors”, and “renal cell carcinoma”. Publications were included in the review if they were including patients with localized RCC. Articles other than English language, editorials, and case reports were excluded.

### 2.2. Assessment of Response

In adjuvant therapeutic investigations, survival endpoints included overall survival (OS), disease-free survival (DFS), and recurrence-free survival (RFS). These terms are defined as the interval of time from randomization to the first recurrence (locally or at distant metastatic sites), or the occurrence of secondary malignancies or death, and are generally used interchangeably [16]. Early investigations tended to focus on RFS as an endpoint, with more recent studies focusing on OS as the primary endpoint [17]. To assess tumor response in neoadjuvant investigations a number of criteria have been utilized to evaluate therapeutic effect: change in tumor size measured in greatest diameter, 2-dimensional product of tumor cross section based on cross-sectional imaging (WHO criteria) [18], Response Evaluation Criteria In Solid Tumors (RECIST) criteria [which defined partial response (PR) as ≥30% reduction in the primary lesion size, progressive disease (PD) as increase in tumor size ≥20% or presence of new lesions or stable disease (SD)] [19], and changes in tumor morphometric score, such as the RENAL (Radius Exophytic Nearness Anterior Location) nephrometry score, a system used for defining tumor complexity [20].  Table 1 demonstrates clinical criteria in which adjuvant and neoadjuvant agents have been investigated. In the adjuvant realm, these have been resected primary tumor and pT2-3 N0 M0 (grades 2-4), pT4 N0 M0, or pTany N1 M0. In the neoadjuvant realm, these are T1-4 NX/1 M0, T1-4 NX/1 M1, borderline resectable masses, facilitation of nephron-sparing surgery, or downstaging IVC thrombi resections.

## 3. Adjuvant Immunotherapy in the Management of Localized and Locally Advanced Renal Cell Carcinoma

In the TKI era, significant investigational efforts were conducted into the utility of these agents as adjuvants after extirpative surgery to reduce risk of recurrence and improve survival, with mixed and largely negative results. A summary of TKI trials is provided in Table 2. The first of these pivotal trials was the ASSURE trial (Adjuvant Sorafenib or Sunitinib in Unfavorable Resected Renal cancer) which enrolled 1943 patients with nonmetastatic high risk RCC with a study design to randomize according to a 1:1:1 ratio to receive Sunitinib 50mg, Sorafenib 800mg, or placebo for 1 year with a primary endpoint of DFS. The study ultimately found no difference in DFS between groups (HR 1.02, 97.5% CI 0.85-1.23) and was hampered by high rates of toxicity and discontinuation in the two treatment arms [36]. The PROTECT trial, which examined two doses of Pazopanib versus placebo, found a marginal benefit in DFS on secondary analysis in those patients receiving higher dose (800mg, HR 0.69 [95% CI 0.51-0.94 p=0.02]), and no difference in the lower dose group [37]. The ATLAS trial compared Axitinib with placebo randomizing 724 patients. The study was closed due to futility as there was no significant difference in DFS (HR=0.87; 95% CI: 0.660-1.147, p=0.321) overall [24]. It was the S-TRAC (Sunitinib Treatment of Renal Adjuvant Cancer) trial that was the first to show a significant improvement in DFS with this class of medications. A total of 615 high risk nonmetastatic patients were randomized to Sunitinib 50mg vs Placebo with a median follow up of 5.4 years. The study demonstrated an improved DFS of 6.8 v 5.6 years, HR 0.76, 95% CI 0.59-0.95, p=0.03. In this, like all the other studies, the toxicity associated with this class of medications was notable, as high as 60.5% [38].

Based on results of the S-TRAC study demonstrating a benefit in DFS, the United States Food and Drug Administration (FDA) approved Sunitinib as an adjuvant agent for high risk localized RCC in November 2017, the first such agent in RCC [15]. Indeed, regulatory approval has heralded a paradigm shift, which has been reflected in the recently updated NCCN guidelines that lists adjuvant therapy with Sunitinib as an option for patients with stage III disease, clear cell histology, and high risk for recurrence [39]. Still, there exist concerns regarding the reproducibility and relatively modest clinical benefit associated with TKI. In February 2018, for example, the European Medicines Agency rejected the use of Sunitinib in the adjuvant setting for high risk localized RCC for these reasons [29]. Nonetheless, enrollment in a clinical trial is still considered a preferred option for most patients at higher risk for recurrence after complete resection for localized RCC.

### 3.1. Immune Checkpoint Inhibitors

The emergence and success of immune checkpoint inhibition as a front-line therapeutic strategy for metastatic RCC has also heralded investigation of these agents as potential adjuvant agents [13, 40]. Indeed, the biologic rationale for immunotherapeutic adjuvant therapy is compelling, and perhaps more so than for TKI agents from a mechanistic standpoint. Clearance of circulating tumor cells or micrometastatic deposits by enhancement of the T1 immune tumor response by blockade of programmed death (PD)-1 receptor and programmed death-ligand 1 (PDL-1) may represent a more efficacious therapeutic pathway that antiangiogenic blockade [21], as has been demonstrated in management of clinical metastatic disease [13, 22]. Currently there are 4 clinical trials examining the potential of checkpoint inhibitors in localized RCC to reduce risk of recurrence: atezolizumab (1 trial, NCT03024996) [40], combination of nivolumab and ipilimumab (1 trial, NCT03138512) [23], pembrolizumab (1 trial, NCT03142334) [30], and durvalumab monotherapy or in combination with tremelimumab (NCT03288532) [41] (summarized in Table 3).

The IMmotion010 trial randomizes resected high risk clear cell or sarcomatoid RCC (pT3a+, high grade including M1 resected disease) to atezolizumab (PDL1 inhibitor) or placebo. The primary end point is RFS determined by central radiologic assessment [40]. Checkmate-914 is a trial enrolling patients to a combination PD1 inhibitor + CTLA4 inhibitor (nivolumab with ipilimumab) or placebo for high risk clear cell RCC [23]. Keynote-564 is enrolling patient for adjuvant pembrolizumab (PD1 inhibitor) verses placebo for high risk patients with clear cell histology including M1 resected disease [30]. The RAMPART study recently began enrolling clear and nonclear cell patients to one of three arms: durvalumab with tremelimumab (PDL1 inhibitor + CTLA inhibitor), or durvalumab monotherapy, or placebo [41]. Current immunotherapeutic ongoing studies in the adjuvant setting are summarized in Table 4.

## 4. Vaccines and Targeted Immunotherapy

Tumor vaccines and targeted immunotherapy have been investigated in the adjuvant setting for RCC. This concept was first explored by Galligioni et al., which utilized autologous tumor cells and bacillus Calmette-Guerin, with negative results [25]. Variations on the same theme have been attempted with the same result [26, 27]. Jocham et al. published results of a randomized trial in 379 patients with pT2-3b N0-3 RCC to receive autologous renal tumor cell vaccine or no treatment and demonstrated decreased tumor progression in the treatment group at 5 years (HR 1.59, CI 1.07 – 2.36, p = 0.0304) [26]. More recently, in the ARISER study, girentuximab, a chimeric antibody targeting carbonic anhydrase IX (CAIX), was evaluated as adjuvant in 864 patients with high risk RCC. Girentuximab was well-tolerated, with toxicity rates comparable to placebo. Overall however, there was no significant difference between girentuximab and placebo for DFS (HR 0.97, 95% CI 0.79–1.18) or OS (HR 0.99, 95% CI 0.74–1.32) [42].

## 5. Neoadjuvant Therapy in Clinically Localized and Locally Advanced Renal Cell Carcinoma

Neoadjuvant therapy for RCC was initially implemented to accomplish reduction in metastatic disease prior to surgical debulking, facilitate more complex surgical resections, and select patients with appropriate disease response to systemic therapy who may benefit from surgical debulking (Table 1) [43, 44]. Indeed, the paradigm of presurgical or primary systemic therapy in the setting of metastatic RCC for TKI has recently been solidified by publication of the SURTIME and CARMENA studies which suggested improvements in PFS with primary TKI prior to surgery and lack of improvement of outcomes in intermediate and high risk metastatic RCC in patients receiving primary cytoreductive nephrectomy, respectively [45, 46]. There have been 15 studies reported in the literature for indications of downstaging tumor size for resection of locally advanced disease (9 studies), facilitating partial nephrectomy (5 studies), and downstaging IVC thrombus level (4 studies). The first study assessing feasibility and efficacy of neoadjuvant therapy prior to resection of locally advanced disease was conducted by Thomas et al. who examined 19 patients with locally extensive primary tumors considered otherwise unresectable were administered Sunitinib (initial dose 50 mg daily) for one 4-week cycle. Analysis noted partial response in 16% (3/19) of patients (by RECIST criteria) with median size reduction of 24% and with 21% (4/19) eventually undergoing nephrectomy. Nonetheless, the authors also reported that 37% of patients experienced grade 3-4 toxicities. No unexpected surgical morbidity was found; however, the major complication rate was not reported [44]. Since then, others that have studied this outcome with various other TKIs have observed 11.8%-28% median reduction in tumor size. In the first prospective randomized double-blind placebo-controlled trial to assess downsizing effect of neoadjuvant TKI, Hatiboglu et al. randomized 12 patients in a 3:1 manner to sorafenib vs placebo and demonstrated median tumor volume reduction of 29% in the treatment group. Nonetheless, toxicity rates are significant as are high grade complications [47–50].

Another indication for investigation into the utility of neoadjuvant therapy has been to facilitate nephron-sparing surgery. The first study to focus on this particular aim was reported by Silberstein et al., who conducted a prospective pilot study and a retrospective multicenter review analyzing outcomes of neoadjuvant Sunitinib (50 mg daily for two 6-week cycles) in 12 patients (14 tumors) with clear cell RCC who had imperative indications for nephron-sparing surgery. The authors noted a mean tumor size reduction of 21.1% (7.1 to 5.6 cm) with 4/14 (28.6%) tumors having PR by RECIST criteria. Ultimately, partial nephrectomy was achievable in all patients without positive margins or requirement for dialysis. Nonetheless, the authors reported that 3/14 (21.4%) renal units experienced urine leaks, all of which resolved with conservative measures [51].

Others have studied the role of various other TKIs in facilitating nephron-sparing surgery and have had mixed results. Taken together the body of work in this area suggests that neoadjuvant TKI therapy for locally advanced disease or prior to partial nephrectomy may result in modest decreases in tumor size and complexity in a subset of patients; partial nephrectomy in this setting remains complex and requires surgical expertise in this area [28, 34, 35, 44, 50–56].

## 6. Neoadjuvant Therapy in the Management of Localized RCC: Future Directions

Similar to the advent of immunotherapeutic investigation for adjuvant therapy in localized RCC, a flurry of high quality studies are currently underway to examine the role of neoadjuvant ICI or combination ICI-TKI targeted therapy for advanced disease, particularly in the wake of the first positive trial demonstrating improved PFS using combination TKI and immune checkpoint inhibitors compared to TKI alone (13.8 vs 7.2 months PFS and response rate of 55.2% vs 25.5% favoring combination therapy) [31]. Currently, seven clinical trials in this arena are ongoing and are summarized in Table 4. Of these studies, 4 involve immune checkpoint inhibitors.

The anti-PD-1 receptor antibody pembrolizumab (1 study; NCT02212730) is currently enrolling (planned accrual of 36 patients) with any RCC histology and clinical cT1b or more, NX-0, M0 disease in a prospective, open label, parallel design [32]^.^ Nivolumab, an anti-PD-1 receptor antibody, is also being studied in the neoadjuvant setting in both clear cell histology and any histology, in several ongoing prospective trials, open label trials, and one randomized double-blind placebo-controlled trial (NCT02575222, NCT02595918, NCT03055013) [33, 57, 58]. Yet another clinical trial involves an antibody directed against programmed cell death-1 ligand 1 (durvalumab/MEDI 4736) ± tremelimumab, an antibody directed against human T-cell receptor protein cytotoxic T-lymphocyte-associated protein 4 (CTLA4). This study is investigating patients with any RCC histology and local or locally advanced disease in a prospective, open label fashion [59]. Finally, an additional clinical trial that evaluated presurgical vaccine therapy was closed after enrolling 4 patients (NCT02170389); further investigation in this region is pending [60].

Utilization of systemic therapy to promote cytoreduction of primary tumors and to facilitate surgical excision should currently be considered to be investigational. Nonetheless, this concept has been borne out in a number of prospective phase II studies [50, 52, 53] and retrospective analyses [54]. The key question, of course, is whether primary systemic therapy facilitates planned surgical intervention that would otherwise not have been feasible. Rini et al. suggested that the partial nephrectomy rate in otherwise unfeasible nephron-sparing situations was 75% [53] and the senior author of the manuscript is the study chair of the largest study to date which will test the question of neoadjuvant therapy prior to imperative indication partial nephrotomy in situations where a partial nephrectomy is not otherwise suitable [61].

A counter argument is often made which is due to variability of surgeon experience and ability, what may be considered unfeasible by one surgeon may indeed be possible and safe by another. While we agree in the validity of this criticism, there nonetheless exists a subset of patients in whom a safe and efficacious nephron-sparing procedure or locally advanced resection is truly not be feasible, and with even mild cytoreduction, feasibility and efficacy of such a resection may be enhanced. The senior author of this manuscript bases his opinion on the fact that he has one of the largest series in the literature of large partial nephrectomies (>7cm) performed, whether by open approach [62, 63] or by minimally invasive approach [64]. We recently also demonstrated efficacy of primary systemic therapy prior to nephrectomy with IVC thrombectomy [65] and believe that our results and those emerging from other groups suggest that concept of primary cytoreductive systemic therapy has merit should be investigated further.

## 7. Conclusion

Utility and efficacy of systemic therapy in the setting of localized and locally advanced RCC are areas of active investigation. Recent approval of Sunitinib as an adjuvant agent has changed the paradigm of management of patients in the United States, and advent of ICI therapy as first line agents for metastatic RCC is spurring further investigation into utility of immunotherapeutic agents or combinations in adjuvant and neoadjuvant settings.

## Figures and Tables

**Table 1:** Clinical criteria for adjuvant therapy or investigations and neoadjuvant therapeutic investigations.

Adjuvant Therapy	Neoadjuvant Therapy
Resected primary tumor	T1-4 NX/1 M0
and	or
pT2-3 N0 M0 (grades 2-4)	T1-4 NX/1 M1
or	or
pT4 N0 M0	Borderline resectable mass
or	or
pTany N1 M0	Facilitating nephron-sparing surgery
	or
	Downstaging IVC thrombus

**Table 2:** Summary of adjuvant trials: completed and reported.

Trial	Design	Intervention	N	Inclusion Criteria (stage/grade/histology)	Results	Adverse Events
ASSURE, Haas et al. (2016) [21]	Randomized, Double-blinded, Placebo-controlled	Sunitinib or Sorafenib	1943	T1b N0 M0 (grade 3-4), pT2–pT4 N0 M0, pT(any) N1 M0; Clear Cell and Non-clear Cell	No difference in median DFS (HR 1.02, 97.5% CI 0.85-1.23)	Grade 3+ toxicities of sunitinib, sorafenib: hypertension (17%, 16%), hand-foot syndrome (15%, 33%), rash (2%, 15%), fatigue (18%, 7%)
PROTECT, Motzer et al. (2017) [22]	Randomized, Double-blinded, Placebo-controlled	Pazopanib	1538	pT2 N0 M0 (grades 3–4), pT3–4 N0 M0, pT (any) N1 M0; Clear Cell	No differences in median DFS (HR 0.86, 95% CI 0.70-1.06)	Increased ALT/AST lead to treatment discontinuation in 600 mg (ALT 16%/AST 5%) and 800 mg (ALT 18%/AST 7%) mg.
ATLAS, Gross-Goupil et al. (2018) [23]	Randomized, Double-blinded, Placebo-controlled	Axitinib	724	pT2–4 N0 M0, pT (any) N1 M0; Clear Cell	No difference in median DFS (HR 0.87, 95% CI 0.66-1.15, p=0.321)	Similar and serious adverse events between groups; more grade 3/4 (61% vs. 30%) for axitinib
S-TRAC, Ravaud et al. (2016) [24]	Randomized, Double-blinded, Placebo-controlled	Sunitinib	615	pT3 N0 M0 (grades 2–4), pT4 N0 M0, pT (any) N1 M0; Clear Cell	Improved median DFS (6.8 years v 5.6; HR 0.76, 95% CI 0.59-0.98)	Increased Grade 3 (48.4% vs. 15.8%); Grade 4 (12.1% vs. 3.6%) in sunitinib; Similar serious event rate.

**Table 3:** Summary of adjuvant and neoadjuvant immunotherapeutic trials: completed and reported.

Trial	Design	Intervention	N	Inclusion Criteria (stage/grade/histology)	Results	Adverse Events
Adjuvant Trials						
Jocham et al. (2004) [25]	Prospective, randomized	Autologous renal tumor cells	558	pT2–3b pN0–3 M0; Clear and Non-Clear Cell	Improved 5 year and 70 month PFS (HR 1.58, 95% CI 1.05-2.37; HR 1.59, 95% CI 1.07-2.36)	Local skin reactions
Wood et al. (2008) [26]	Prospective, randomized	Autologous tumor-derived protein	819	cT1b–4 N0 M0, cT(any) N1-2 M0; Clear and Non-Clear Cell	No difference in PFS at 1.9 median year follow-up (HR 0.92, 95% CI 0.729-1.169)	Local skin reactions
ARISER, Chamie et al. (2016) [27]	Randomized, Double-blinded, Placebo-controlled	Girentuximab	864	pT1b–2 (Fuhrman ≥3), pT3–4 N0, pT(any) N+; Clear Cell	No difference in DFS (HR 0.97, 95% CI 0.79-1.18) or OS (HR 0.99, 95% CI 0.74-1.32	Toxicity rate 21%, comparable to placebo
Neoadjuvant Trial						
Cost et al (2011) [28]	Retrospective	Sunitinib (12), bevacizumab (9), sorafenib (1), temsirolimus (3)	25	T3b+M1 (21)	25/0	12% downstage thrombus level; 4% upstage level; 4% altered surgical strategy

**Table 4:** Summary of adjuvant and neoadjuvant studies: ongoing or unreported.

Trial	Design	Agent	Planned Accrual	Inclusion Criteria (stage/grade)	Inclusion Criteria (histology)
Adjuvant Trials					

IMmotion010, (NCT03024996) [29]	Prospective, double-blinded, placebo controlled	Atezolizumab	664	Nonmetastatic	Clear cell, sarcomatoid

Checkmate-914, (NCT03138512) [22]	Prospective, double-blinded, placebo controlled	Nivolumab + Ipilimumab	800	pT2a – 4 N0 M0 (any), pT1-4 N1 M0 (any)	Clear cell

Keynote-564, (NCT03142334) [23]	Prospective, double-blinded, placebo controlled	Pembrolizumab	950	pT2 N0 M0 (grade 4 or sarcomatoid), pT3-4 N0 M0 (any), pT1-4 N1 M0, Resectable M1	Clear cell

RAMPART, (NCT03288532) [30]	Prospective, multicenter, double-blinded, placebo controlled	Durvalumab, Durvalumab + tremelimumab	1750	Leibovich Score 3-11	Any

Neoadjuvant Trials					

Merck Sharp Dohme Corp (NCT02212730) [31]	Prospective, open label, parallel assignment	Pembrolizumab	36	cT1b+ NX-0 M0	Any

Bristol-Myers Squibb (NCT02575222) [32]	Prospective, open label	Nivolumab	30	cT2a-T4 NX-1 M0, cT1-4 N1 M0	Clear cell

NCI (NCT02595918) [33]	Prospective, open label	Nivolumab	29	Stage I-III	Clear cell

Case Comprehensive Cancer Center (NCT02762006) [34]	Prospective, open label	Durvalumab, Tremelimumab	45	cT2b-4 NX-0 M0 cT1-4 N1, M0	Any

PROSPER, (NCT03055013) [35]	Randomized, double-blind, placebo controlled	Nivolumab	766	cT2 NX M0, cT1-4 N1 M0	Any

Roswell Park Cancer Institute (NCT02170389) [31]	Prospective, open label	RCC/CD40L RNA-Transfected Autologous Vaccine	4	pT1, NX-0, M0	Any
